# Polyethylene Glycol Camouflaged Earthworm Hemoglobin

**DOI:** 10.1371/journal.pone.0170041

**Published:** 2017-01-18

**Authors:** Vivek P. Jani, Alborz Jelvani, Selamawit Moges, Parimala Nacharaju, Camille Roche, David Dantsker, Andre Palmer, Joel M. Friedman, Pedro Cabrales

**Affiliations:** 1 Bioengineering, University of California San Diego, La Jolla, California, United States of America; 2 Medicine, Albert Einstein College of Medicine, Bronx, New York, United States of America; 3 William G. Lowrie Department of Chemical and Biomolecular Engineering, The Ohio State University, Columbus, Ohio, United States of America; Albany Medical College, UNITED STATES

## Abstract

Nearly 21 million components of blood and whole blood and transfused annually in the United States, while on average only 13.6 million units of blood are donated. As the demand for Red Blood Cells (RBCs) continues to increase due to the aging population, this deficit will be more significant. Despite decades of research to develop hemoglobin (Hb) based oxygen (O_2_) carriers (HBOCs) as RBC substitutes, there are no products approved for clinical use. *Lumbricus terrestris* erythrocruorin (LtEc) is the large acellular O_2_ carrying protein complex found in the earthworm *Lumbricus terrestris*. LtEc is an extremely stable protein complex, resistant to autoxidation, and capable of transporting O_2_ to tissue when transfused into mammals. These characteristics render LtEc a promising candidate for the development of the next generation HBOCs. LtEc has a short half-life in circulation, limiting its application as a bridge over days, until blood became available. Conjugation with polyethylene glycol (PEG-LtEc) can extend LtEc circulation time. This study explores PEG-LtEc pharmacokinetics and pharmacodynamics. To study PEG-LtEc pharmacokinetics, hamsters instrumented with the dorsal window chamber were subjected to a 40% exchange transfusion with 10 g/dL PEG-LtEc or LtEc and followed for 48 hours. To study the vascular response of PEG-LtEc, hamsters instrumented with the dorsal window chamber received multiple infusions of 10 g/dL PEG-LtEc or LtEc solution to increase plasma LtEc concentration to 0.5, then 1.0, and 1.5 g/dL, while monitoring the animals’ systemic and microcirculatory parameters. Results confirm that PEGylation of LtEc increases its circulation time, extending the half-life to 70 hours, 4 times longer than that of unPEGylated LtEc. However, PEGylation increased the rate of LtEc oxidation *in vivo*. Vascular analysis verified that PEG-LtEc showed the absence of microvascular vasoconstriction or systemic hypertension. The molecular size of PEG-LtEc did not change the colloid osmotic pressure or blood volume expansion capacity compared to LtEc, due to LtEc’s already large molecular size. Taken together, these results further encourage the development of PEG-LtEc as an O_2_ carrying therapeutic.

## Introduction

Both in the United States (US) and globally, the need for red blood cell (RBC) transfusions has steadily increased over the years.[1] Studies indicate that the demand for RBCs in the US relative to the amount collected will create a shortage of 3 million units of RBCs by the year 2030.[1] This shortage of RBCs is highest in sub-Saharan Africa, where the demand for RBCs is double the amount of RBCs collected from donors.[2] According to the World Health Organization (WHO), 20% of deaths due to malaria and 40% of deaths during childbirth in third-world countries are related to an inadequate blood supply.[3] Since sophisticated blood screening techniques are available in the US, disease transmission after transfusion is rare; however, according to WHO, in some third world countries, including those in Africa, there is a 95–100% chance of human immunodeficiency virus (HIV) contraction after transfusion of unmonitored and unsafe allogeneic blood.[4] Even in the US, the chance of bacterial contamination through RBC transfusion is still high (1 in 38,500).[5, 6] It has also been reported that transfusion of RBCs may increase the risk of transmission of hepatitis, variant Creutzfeldt-Jakob disease, West Nile virus, Zika virus, and internal systemic complications such as immune system suppression, acute lung injury, and allergic reactions. Additionally, RBC accessibility during emergency scenarios and on the battlefield, is extremely limited due to the short shelf-life, and required storage conditions for RBCs. In these conditions, 25% of all trauma casualties are transfused with an average of 1–4 units of RBCs.[7] At least 80% of casualties, who could potentially survive, die from an inadequate supply of RBCs and exsanguinating hemorrhage.[8] The growing demand for RBCs along with the aforementioned issues associated with RBC transfusion justify the need for alternatives to RBCs, that have greater availability, accessibility, shelf life, oxygen (O_2_) transport capacity, and safety.

Over the last few decades, artificial O_2_ carriers such as perfluorocarbon (PFC) emulsions and hemoglobin (Hb) based O_2_ carriers (HBOCs) have been developed as potential RBC substitutes.[9] PFCs and HBOCs have reached clinical trials; however, short circulation time and adverse side-effects, including stroke, multi-organ failure, myocardial infarction, acute respiratory distress syndrome, and even death, have resulted in termination of further development.[10] The chemical modifications of Hb necessary to synthesize HBOCs generally increase the autoxidation rate of Hb and consequently oxidative tissue injury.[11] Some of the adverse side-effects previously observed with HBOCs have been attributed to Hb autoxidation, extravasation through the blood vessel wall, nitric oxide (NO) scavenging, and hyper-oxygenation of the vessel wall (i.e. O_2_ autoregulation).[9] Vasoconstriction is perceived to be the most critical barrier hampering the development of HBOCs. HBOC-induced vasoconstriction has been attributed to NO scavenging by acellular Hb via either NO binding to deoxyHb, resulting in the highly stable ferrous nitrosyl Hb derivative, or through the NO dioxygenase reaction between NO and oxygenated Hb (oxyHb) to yield methemoglobin (metHb) and nitrate.

Lumbricus Terrestris erythrocruorin (LtEc) has naturally adapted to limit the problems associated with acellular HBOCs [12]. Hirsch *et al*. previously have shown that LtEc when injected into mice and rats produced no noticeable side effects [12]. LtEc has been studied before as an O_2_ carrier because it has a lower rate of oxidation [redox potential, Eo = 112 mV] compared to mammalian Hbs [Eo = -50 mV for HbA] [13]. Additionally, LtEc and other erythrocruorins have an extremely low rate of NO scavenging [14]. LtEc is a complex structure, containing 144 globin subunits and 36 linker proteins. The complex consists of five types of globins [A, B, C, and D1 or D2] and four types of linkers [14]. Each globin contains a heme group and binds O_2_. Each subunit assembles into an ABC trimer via intermolecular disulfide bonds. The trimers pairs with D monomers to form the ABCD tetramers through electrostatic and hydrophobic interactions, which then associate with two more tetramers via linkers to form dodecamers. Finally, twelve dodecamers assemble into the hexagonal bilayer structure, which has a MW of 3.6 MDa and a molecular radius of approximately 15 nm. As LtEc is a relatively large and stable molecule, it is difficult to purify; however, to overcome this limitation, LtEc can been purified by tangential-flow filtration (TFF). In addition to TFF being scalable, is a reliable process with high yield (grams from 1k worms) and with relatively low costs [14].

Recently, we have shown that *Lumbricus Terrestris* erythrocruorin (LtEc) can be used as a HBOC in small animals.[14, 15] LtEc has been shown to have a circulation time of 13–17 hours *in vivo*. Transfused RBCs remain in circulation and continue to function for several days and even up to weeks; the same, however, cannot be said about HBOCs. It is therefore a significant challenge to formulate HBOCs that match the efficacy and functionality of RBCs. An optimal HBOC should have a persistent circulation time coupled with a long-functional half-life to transport and deliver O_2_, in order to reduce the frequency of administration of the HBOC until either blood for transfusion is available or erythropoiesis replenishes the circulating RBCs. LtEc’s short circulation time is a serious limitation in its use as an O_2_ ‘bridge’ during anemia. This study evaluates a strategy to increase LtEc circulation time based on polyethylene glycol (PEG) surface conjugation to purified LtEc. PEGylation is a common method used to decrease excretion, extend circulation time, and limit immune recognition of proteins in the circulation.[16] This study aims to the test whether PEGylated LtEc (PEG-LtEc) increases circulation half-life, while maintaining LtEc favorable properties and preserving reduced vasoactivity. The pharmacokinetics of PEG-LtEc was studied for 48 hours after an exchange transfusion of 40% of the animal’s blood volume (BV) with 10 g/dL PEG-LtEc solution. The vascular response of PEG-LtEc was studied via infusion of a 10 g/dL PEG-LtEc solution to increase consecutively PEG-LtEc plasma concentration to 0.5, then 1.0, and lastly 1.5 g/dL, while monitoring the animals’ systemic and microcirculatory parameters. UnPEGylated LtEc was used as a control. Vasoactivity of LtEc and PEG-LtEc was assessed in the blood vessels most sensitive to the well accepted mechanism of vasoactivity by HBOCs (NO scavenging by heme, O_2_ over supply by acellular Hb, and Hb extravasation). These microvascular blood vessels have diameters below 80 micrometers, and their tunica media is made up mostly of smooth muscle fibers arranged around the vessel to maximally regulate blood flow in the microcirculation. The larger arteries, however, do not actively change diameter, as changes in these arteries would likely result in drastic changes in blood flow downstream. Our results demonstrate that PEG-LtEc circulated four times longer that of LtEc with no observable vasoconstriction and hypertension, while preserving microvascular blood flow and functional capillary density (FCD).

## Material and Methods

### Earthworm Preparation

For each round of LtEc purification, 1,000 Canadian nightcrawlers (*Lumbricus terrestris*) were purchased from Wholesale Bait Company (Hamilton, OH). Worms were rinsed in tap water to remove dirt, and 1 L batches of worms were extensively washed with 20–30 L of tap water to remove as much mucus as possible. A blender was used to homogenize the worms (puree mode for ~10 seconds) and the homogenate was immediately centrifuged at 3,716 g for 40 minutes at 4°C. Solid debris was discarded and the cloudy red supernatant was centrifuged at 18,000 g for 20 minutes at 10–15°C. The clear red supernatant (~2.0 L per 1,000 worms) was then filtered through to remove any remaining large particles.

### Purification of LtEc

The earthworm homogenate was passed through two 0.22 μm tangential flow filtration (TFF) cartridges in parallel (1050 cm^2^ surface area, Spectrum Labs, Rancho Dominguez, CA) at 450 mL/min until most the sample volume was transmitted through the membrane. The 0.22 μm filtrate (1.8–2.0 L) was then concentrated on two 500 kDa TFF cartridges (1050 cm^2^ surface area, Spectrum Labs) at 450 mL/min down to a final volume of approximately 200 mL. The 500 kDa retentate was then diafiltered by diluting it to 2 L with buffer and then concentrating it down to 200 mL ten times. The retentate was first diluted with 20 mM Tris buffer (pH 7.0) during the first eight rounds of diafiltration and a modified lactated Ringer’s buffer (115 mM NaCl, 4 mM KCl, 1.4 mM CaCl_2_, 13 mM NaOH, 12.25 mM *N*-acetylcysteine, 0.3% sodium lactate, pH 7.0) during the last two rounds of diafiltration. During the final round of diafiltration, the retentate was concentrated to 20–50 mL and sterilized by passing it through a 0.45 μm syringe filter. The purified Ec was then stored at -80°C until needed. After each round of purification, all filters were rinsed and soaked in 0.2 M NaOH for 1 hour, then rinsed with distilled water and stored at 4°C.[15]

### PEGylation of LtEc

LtEc (75 mg/ml) was incubated with 5 mM 2-iminothiolane and 10 mM maleimide-PEG-5000 in PBS, pH 7.4 at 4°C overnight. After incubation, excess (unreacted) reagents were removed by filtering the reaction mixture through 50 kDa membrane filters (Millipore) by repeated dilution/centrifugation. The removal of the PEG reagent was monitored by size exclusion chromatography of the reaction mixture using a refractive Index detector.[17] The final product of PEGylated LtEc (PEG-LtEc) was concentrated to 100 mg/ml for animal studies.

### Viscosity and Colloid Osmotic Pressure

Viscosity was measured at a shear rate of 160/sec (Brookfield Engineering Laboratories, Middleboro, MA). Colloid osmotic pressure (COP) was measured using a 4420 Colloid Osmometer (Wescor, Logan, UT).

### Mass Spectral Analysis

Samples were prepared for Matrix Assisted Laser Desorption Ionization (MALDI) by diluting LtEc and PEG-LtEc to 1 mg/mL, then mixing them in a 1:1:1 ratio with 1M HCl and a matrix solution of 50% v/v acetonitrile saturated with sinapic acid [18]. Cytochrome C (12,361 g/mol), apomyoglobin (16,952 g/mol), and human serum albumin (66,437 g/mol) were used as molecular weight (MW) standards on a Bruker FLEX MALDI-TOF (Bruker Daltonics, Billerica, MA) and analyzed in the linear mode [18].

### Oxygen equilibrium Curve (OEC)

OECs were collected using the TCS Hemox Analyze (TCS Scientific Company, New Hope, PA). Briefly, approximately 20–200 μL of Hb sample (final concentration ~5 mg/mL) was diluted with 4.9 mL of Hemox Buffer (pH 7.4). 20μL of Additive A (albumin), 10 μL of Additive B, and 10 μL of an anti-foaming agent (TCS Scientific) were added to the solution. During deoxygenation the O_2_ tension and %O_2_ saturation are recorded to obtain an OEC, and P50 value (partial pressure of O_2_ at which Hb is 50% saturated with O_2_). The Hemox analyzer software was used to calculate P50 and cooperativity based on O_2_ binding.

### Nitrite Reductase

Nitrite reductase reactions were performed in parallel (HbA, LtHb and PEG-LtEc) using the argon-purged deoxy heme at 0.20 mM in the presence of 1 mM dithionite. Changes in the deoxy heme Soret 430 nm band were recorded as a function of time (Lambda 20 UV/VIS spectrometer; Perkin Elmer, Foster City, CA). Under these conditions, the nitrite reductase reaction is modified, since the metHb generated from the reaction is reduced back to deoxyHb by the excess dithionite. The deoxy-Hb binds NO, yielding the ferrous NO derivative of Hb (NOHb). This approach is based on the relatively large extinction coefficient for deoxy heme (@ 430nm), as compared to the smaller extinction coefficients of both NOHb and metHb. The initial linear portion of the curve was used to calculate the initial reaction rates.

### Animal Preparation

In vivo studies were performed in 54–66 g male Golden Syrian Hamsters (Charles River Laboratories, Boston, MA) fitted with a dorsal skinfold window chamber. The hamster window chamber model is widely used for microvascular studies in the unanesthetized state (complete surgical technique is described in the literature).[19, 20] Arterial and venous catheters filled with a heparinized saline solution (30 IU/ml) were implanted into the carotid and jugular vessels. Catheters were tunneled under the skin, exteriorized at the dorsal side of the neck, and securely attached to the window frame. Animal handling and care followed the NIH Guide for the Care and Use of Laboratory Animals. The experimental protocol was approved by UC San Diego Animal Care and Use Committee (IACUC).

### Inclusion Criteria

Animals were considered suitable for experiments if systemic parameters were within normal range, namely, heart rate (HR) > 340 beats/min, mean arterial blood pressure (MAP) > 80 mmHg, systemic Hct > 45%, and arterial O_2_ partial pressure (PaO_2_) > 50 mmHg. Additionally, animals were examined 3 to 4 days after implantation surgery, their tissue was observed under 650× magnification, and only animals without signs of low perfusion, inflammation, edema or bleeding were included in the study.

### Systemic Parameters

MAP and HR were recorded continuously (MP 150, Biopac System, Santa Barbara, CA). Hct levels were measured from centrifuged arterial blood samples taken in heparinized capillary tubes. Hb content was determined spectrophotometrically (B-Hemoglobin, Hemocue).

### Blood Chemistry and Biophysical Properties

Arterial blood was collected in heparinized glass capillaries (50 μL) and immediately analyzed for pO_2_, pCO_2_, base excess, and pH (Rapidlab 248, Bayer, Norwood, MA).

### Estimation of Yield and MetHb Level

LtEc concentration and the percent of oxidized LtEc and PEG-LtEc (i.e. met-LtEc and met-PEG-LtEc) were measured using the cyanomethemoglobin method.[21]

### Volume Expansion

Changes in blood volume was calculated as the difference in Hct before infusion (Hct_0_) and the Hct at a specific time after infusion (Hct_t_):

$$V_{E}=\frac{{Hct}_{0}-{Hct}_{t}}{{Hct}_{t}}$$

### Experimental Groups

Experimental groups were labeled based on the test solution infused and included LtEc and PEG-LtEc test solutions.

### Pharmacokinetics—Exchange Transfusion Protocol

Pharmacokinetics was studied for 48 hours after exchange transfusion with 10 g/dL PEG-LtEc or LtEc solution. Briefly, hamsters fitted with the dorsal window chamber were exchanged with 40% of the estimated BV (7% of body weight). Test solutions were infused into the jugular vein catheter at a rate of 100 μl/min with simultaneous blood withdrawal at the same rate from the carotid artery catheter via a dual syringe pump (Harvard Apparatus, Holliston, MA). Blood samples (50 μL) were taken after exchange transfusion at 0.5, 1, 2, 4, 8, 12, 24, and 48 hours. PEG-LtEc and LtEc pharmacokinetic parameters were determined using non-compartmental analysis.

### Vascular Responses—Hypervolemic Infusion (Top-Load) Protocol

PEG-LtEc and LtEc vascular responses were studied after infusion of 10 g/dL PEG-LtEc or LtEc solution to increase protein plasma concentration. Hamsters fitted with the dorsal window chamber were randomly divided into two experimental groups. Three consecutive infusions of the test solutions were administered to increase the plasma protein concentrations to 0.5, 1.0, and ultimately 1.5 g/dL. All infusions were performed intravenously at a rate of 100 μL/min. The plasma Hb concentration was determined spectrophotometrically (B-hemoglobin, Hemocue, Stockholm, Sweden). Infusion volumes of each test solution required to increase the plasma protein concentration by 0.5 g/dL were estimated in each animal before infusion, based on the estimated animal BV and Hct, as 0.05 × (1 − Hct) × BV. Five minutes after infusion, the plasma protein concentration was verified and increased if necessary. After each infusion, animals were allowed 30 to 40 minutes to stabilize before systemic and microvascular characterization.

### Microvascular Experimental Setup

The unanesthetized animal was placed in a restraining tube with a longitudinal slit from which the window chamber protruded and was then fixed to the microscopic stage for transillumination with a customized intravital microscope (BX51WI, Olympus, New Hyde Park, NY). Animals were given 20 minutes to adjust to the tube environment before any measurements were made. The tissue image was projected onto a charge-coupled device camera (4815, COHU, San Diego, CA) connected to a video recorder and viewed on a monitor. Measurements were carried out using a 40× (LUMPFL-WIR, numerical aperture 0.8, Olympus) water immersion objective.

### Functional Capillary Density

Functional capillaries (defined as those capillary segments that exhibit RBC transit of at least a single RBC in a 45-second period in 10 successive microscopic fields) were assessed in a region measuring 0.46 mm^2^. The relative change in functional capillary density (FCD) from baseline levels after each infusion is indicative of the extent of capillary perfusion.[22]

### Microhemodynamics

Arteriolar and venular blood flow velocities were measured online by using the photodiode cross-correlation method (Vista Electronics, San Diego, CA).[23] The measured centerline velocity (V) was corrected according to blood vessel size to obtain the mean RBC velocity (V/R_v_), where R_v_ represents the ratio between the blood vessel centerline V and the blood vessel mean blood V based on data obtained in glass tubes. According to Lipowsky and Zweifach, R_v_ = 1.6 for blood vessels between 15 and 90 μm diameter, but not for larger blood vessels.[24] A video image-shearing method was used to measure blood vessel diameter D (Vista Electronics, San Diego, CA).[25] The blood flow rate (Q) was calculated from measured values as:

$$Q=\pi \times \left(\frac{V}{R_{v}}\right)\times {\left(\frac{D}{2}\right)}^{2}$$

### Statistical Analysis

Results are presented as the mean ± standard deviation. Data within each group were analyzed using analysis of variance for repeated measurements (ANOVA, Kruskal-Wallis test). When appropriate, post hoc analyses were performed with the Dunn’s multiple comparison test. Comparison between samples was performed using two-way ANOVA (Hb plasma concentration); post hoc analyses were performed with Bonferroni posttests. Microhemodynamic data are presented as absolute values and ratios relative to baseline values. A ratio of 1.0 signifies no change from baseline, while lower and higher ratios are indicative of changes proportionally lower and higher than baseline (i.e., 1.5 represents a 50% increase from the baseline level). The same blood vessels and capillary fields were monitored throughout the study, such that direct comparisons to their baseline levels could be performed, allowing for more reliable statistics on small sample populations. All statistics were calculated using computer software (GraphPad Prism 6, GraphPad Software, Inc., San Diego, CA). Changes were considered significant if *p* values were less than 0.05.

## Results

### Oxygen Binding and Biophysical Properties

Summary of O_2_ binding and biophysical characteristics are summarized in **Table 1.** PEGylation of LtEc had no statistically significant effect on O_2_ affinity, (P50, O_2_ tension at which Hb is 50% saturated with O_2_). However, PEGylation statistically decreased cooperativity of LtEc, suggesting that PEG reduces subunit conformational switch from a weak to strong O_2_ binding state and limits the interactions between subunits. PEGylation also resulted in statistically significant increases in both viscosity and colloid oncotic pressure (COP) relative to LtEC.

**Table 1:** Properties of LtEc and PEG-LtEc.

Properties	LtEc				PEG-LtEc		
P50, mmHg	26.0	±	1.2		25.2	±	1.4
Cooperativity	2.4	±	0.3		1.9	±	0.2†
MetHb, %	4.6	±	1.8		4.1	±	1.6
COP, mmHg	1.0	±	0.2		3.8	±	0.3†
Viscosity, cP	1.3	±	0.1		1.9	±	0.1

Results obtained from three (3) different batches of purification and PEGylation.

†, P<0.05 compared to LtEc

### Mass Distribution

**Fig 1:**
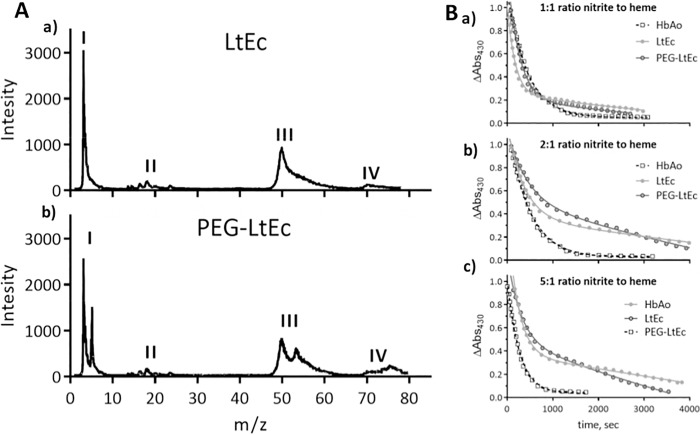
**A. MALDI mass spectrum.** a) LtEc (top) and b) PEG-LtEc (botton), MW range of 10–80 kDa. I) monomers; II) linkers; III) trimers (A, B, and C subunits); IV) tetramers (A, B, C, and D subunits). **B. Nitrite reductase at pH 7.0 monitored as the decay of the 430 nm Soret band as a function of the ratio nitrite to heme.** a) 1:1; b) 2:1; c) 5:1. Heme concentration in all cases was 0.20 mM. The lines represent fits to the data. The traces were all normalized for comparison of protein kinetics.

The MALDI mass spectrum of LtEc and PEG-LtEc (Fig 1A) show similar to LtEc mass spectra existing in the literature [18]. For LtEc, monometers and linkers appear near their expected MW (16 kDa and 24–32 kDa), respectively. While trimers appear to have a MW of around 50 kDa and tetramers a MW near 70 kDa. PEGylation affects the molecular weights of the subunits, as it shifts the MW by 5 and 10 kDa for monomers and trimers and tetramers, respectively. However, the native mass spectrum signature of the unPEGylated subunits remains in the mass spectrum of PEG-LtEc, suggesting that only a fraction of the subunits was PEGylated and another fraction remains unPEGylated. Additionally, it is important to note, that no major impurities were detected in the MALDI mass spectrum.

### Nitrite Reductase

Nitrite reductase at various nitrite to heme ratios for HbA, LtEc and PEG-LtEc are shown in Fig 1B. The normalized nitrite-induced decay of the deoxyheme population as a function of the ratio of nitrite to heme for LtEc and PEG-LtEc appears to exhibit two distinct phases, whereas HbA only exhibits one phase. LtHb does not undergo any R/T allosteric transitions; hence the two distinct phases for LtHb and PEG-LtEc can be attributed to the fact that there are two different heme populations. The first phase and fast nitrite reductase rates at a 1:1 nitrite to heme ratio were 0.142, 0.068 and 0.076 μM/s for HbA, LtEc and PEG-LtEc respectively. The initial fast nitrite reductase rate increased for HbA with the nitrite to heme ratios. However, for LtEc and PEG-LtEc, the initial fast nitrite reductase rate remained constant and the second slower nitrite reductase rate phase decreased with higher ratios of nitrite to heme.

### Pharmacokinetics of LtEc and PEG-LtEc

Pharmacokinetics results are summarized in **Table 2**. The mean residence time (MRT) and half life (t_1/2_) were statistically higher (P<0.05) for PEG-LtEc as compared to LtEc. Additionally, the terminal K (terminal slope) and clearance were found to be statistically lower (P<0.05) after PEGylation. The maximum plasma concentration (C_max_) and volume of distribution at steady state (V_ss_) were found to be statistically higher (P<0.05) after PEGylation. Overall, PEG-LtEc has an improved circulatory persistence, found to be 4 times greater than that of LtEc.

**Table 2:** Pharmacokinetics of LtEc and PEG-LtEc.

	LtEc			PEG-LtEc		
Terminal *K*, h^-1^	0.04	±	0.02	0.01	±	0.01^†^
Half life, h	18.0	±	0.8	66.8	±	1.8^†^
CL, mL.h^-1^	3.82	±	0.08	0.84	±	0.06^†^
MRT, h	23.4	±	0.9	95.7	±	1.9^†^
C_max_, mg.h^-1^	2.85	±	0.2	3.12	±	0.22
V_ss_, mL	23.5	±	1.1	80.6	±	2.4^†^

Terminal K, terminal slope; CL, plasma clearance; MRT, mean residence timel; Cmax, concentration maximal; Vss, volume of distribution at steady state.

†, P<0.05 compared to LtEc

**Fig 2:**
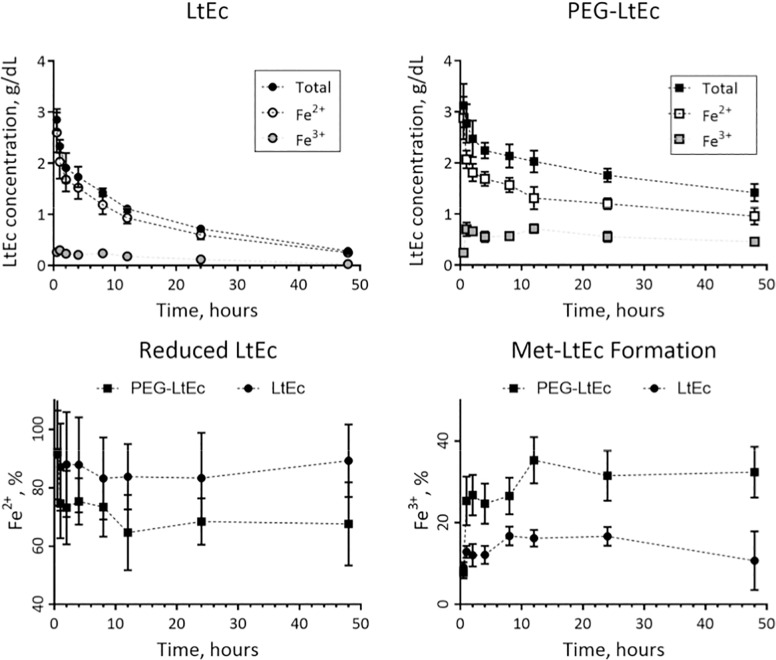
LtEc and PEG-LtEc pharmacokinetics after a 40% exchange transfusion (Top panels). The concentration of the reduced (Fe^2+^) and oxidized (Fe^3+^) forms of PEG-LtEc and LtEc as a function of time (bottom panels).

The concentration of the reduced (Fe^2+^) and oxidized (Fe^3+^) forms of PEG-LtEc and LtEc as a function of time after exchange transfusion are displayed in Fig 2. The reduced PEG-LtEc concentration was found to be statistically different (P<0.05) compared to the reduced LtEc after 24 hours of circulation time, while oxidized PEG-LtEc was found to be statistically different (P<0.05) compared to oxidized LtEc only after 1 hour of circulation time. The formation of oxidized Fe^3+^ LtEc (i.e. metHb) was found to be statistically different (P<0.05) between PEG-LtEc and LtEc after 1 hour of circulation time, while formation of reduced metHb was found to be statistically different (P<0.05) between PEG-LtEc and LtEc after 12 hours of circulation time. The functional fraction to transport and deliver O_2_ to tissue was overall reduced by PEGylation of LtEc. However, the optimal increases in O_2_ carrying capacity requires both a long circulation time coupled with functional half-life. Thus, the half-life of reduced forms of PEG-LtEc and LtEc, is the appropriate parameter to compare improvements in O_2_ transport. This study observed that 54 ± 5% of the administered PEG-LtEc remained functional to transport O_2_ at 8 hours, while only 41 ± 6% of the administered LtEc remained functional to transport O_2_ at 8 hours.

### Pharmacodynamics of PEG- LtEc and LtEc

**Fig 3:**
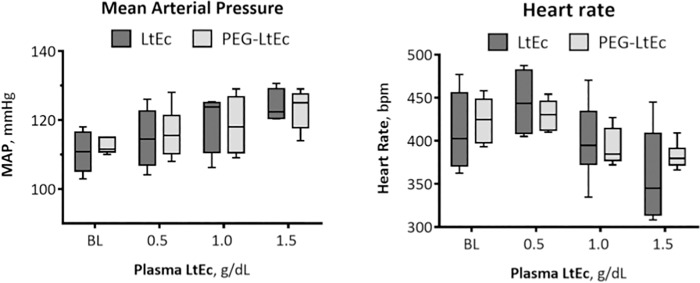
Mean arterial pressure (MAP) and heart rate (HR) after hypervolemic infusion, of LtEc or PEG-LtEc. †, P < 0.05 relative to baseline; ‡, P < 0.05 compared to LtEc. During the hypervolemic infusion, each animal was infused with enough material to increase its plasma protein concentration to 0.5 g/dL, then given 30 minutes to recover. This process was repeated twice, ones to increase its protein concentration to 1.0 g/dL and then to 1.5 g/dL.

MAP and HR after infusion of PEG-LtEc and LtEc are presented in Fig 3. No statistical differences were observed for both MAP and HR between LtEc and PEG-LtEc.

### Hematological and Blood Gas Parameters

Hct and total Hb were found to be statistically lower (P<0.05) for PEG-LtEc compared to LtEc at a plasma protein concentration of 1.5 g/dL. No statistical differences were observed at lower plasma protein concentrations. These results are summarized in **Table 3**. Additionally, no statistical differences in arterial blood pH, arterial blood pO_2_, and arterial blood pCO_2_ were observed after infusion of PEG-LtEc and LtEc at all plasma concentrations (**Table 4**).

**Table 3:** Effects of consecutive transfusions of LtEc, and PEG-LtEc on Hct, total Hb concentration, and plasma protein concentration.

Parameters	LtEc	PEG-LtEc
n	6	6
Baseline		
Hct (%)	49 ± 1	49 ± 1
Hb (g/dL)	15.5 ± 0.4	15.7 ± 0.6
Body weight (g)	60.8 ± 2.0	61.9 ± 4.4
0.5 g/dL		
Volume infused (mL)	0.096 ± 0.003	0.100 ± 0.006
Hct (%)	47 ± 1	46 ± 1
Hb_total_ (g/dL)	14.6 ± 0.5	14.6 ± 0.4
Protein_plasma_ (g/dL)	0.5 ± 0.1	0.5 ± 0.1
Volume Expansion %	4 ± 2	6 ± 3
1.0 g/dL		
Volume infused (mL)	0.198 ± 0.005	0.205 ± 0.959
Hct (%)	44 ± 1	43 ± 1
Hb_total_ (g/dL)	13.8 ± 0.5	13.5 ± 0.7
Protein_plasma_ (g/dL)	1.1 ± 0.2	1.0 ± 0.1
Volume Expansion %	8 ± 1	7 ± 4
1.5 g/dL		
Volume infused (mL)	0.302±0.005	0.315 ± 0.961
Hct (%)	43 ± 1	41 ± 1^†^
Hb_total_ (g/dL)	13.2 ± 0.3	12.5 ± 0.3^†^
Protein_plasma_ (g/dL)	1.5 ± 0.1	1.5 ± 0.1
Volume Expansion %	3 ± 1	5 ± 3

^†^, P<0.05 compared to LtEc

**Table 4:** Changes in blood gas parameters after consecutive infusions of LtEc or PEG-LtEc.

Parameters	LtEc	PEG-LtEc
n	6	6
Baseline		
pH	7.351 ± 0.015	7.355 ± 0.009
pO_2_, mmHg	72.8 ± 4.1	73.3 ± 4.1
pCO_2_, mmHg	46.3 ± 3.1	44.5 ± 2.3
0.5 g/dL		
pH	7.349 ± 0.018	7.345 ± 0.023
pO_2_, mmHg	74.1 ± 3.9	73.1 ± 3.7
pCO_2_, mmHg	43.6 ± 3.7	43.5 ± 1.4
1.0 g/dL		
pH	7.342 ± 0.022	7.342 ± 0.011
pO_2_, mmHg	76.8 ± 3.6	75.7 ± 3.7
pCO_2_, mmHg	43.4 ± 5.2	42.6 ± 2.0
1.5 g/dL		
pH	7.340 ± 0.021	7.343 ± 0.013
pO_2_, mmHg	77.2 ± 5.4	76.5 ± 2.4
pCO_2_, mmHg	43.1 ± 4.3	42.5 ± 1.9

### Microvascular Hemodynamics

Blood vessel diameter and blood flow were measured for arterioles and venules with diameters between 20 and 80 μm.

#### 20–40 μm Arterioles

**Fig 4:**
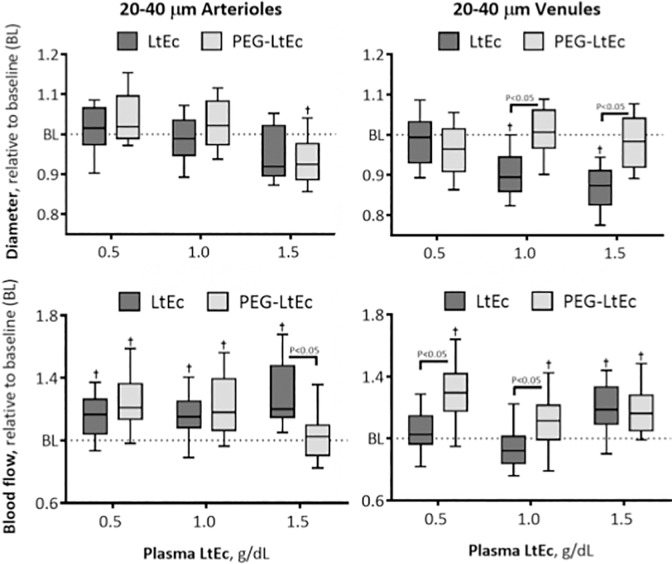
Changes in diameter and blood flow after hypervolemic infusion, of LtEc or PEG-LtEc, for small arterioles and venules with diameters at baseline between 20–40 μm. †, P < 0.05 relative to baseline; ‡, P < 0.05 compared to LtEc. Vessel diameter and blood flow were studied at plasma protein concentration to 0.5, 1.0, and 1.5 g/dL.

Changes in 20–40 μm arterioles diameter at various Hb concentrations are shown in Fig 4. A statistically significant decrease in blood vessel diameter and increase blood flow were observed from baseline blood vessel diameters at a plasma concentration of 1.5 g/dL and from baseline blood flow at all plasma concentrations greater than or equal to 0.5 g/dL after infusion of LtEc. Infusion with PEG-LtEc, however, resulted in statistically significant decrease from baseline blood vessel diameters at all plasma concentrations greater than or equal to 0.5 g/dL and statistically significant increase from baseline blood flow at plasma concentrations less than 1.5 g/dL. No statistical differences were observed in blood vessel diameters between the two groups; however, blood flow was found to be statistically lower after infusion of PEG-LtEc compared to LtEc.

#### 20–40 μm Venules

Changes in 20–40 μm venules diameter at various Hb concentrations are shown in Fig 4. Statistically significant decrease in blood vessel diameter from baseline were observed at plasma concentrations greater than 0.5 g/dL, while statistically significant increases in blood flow from baseline were observed at all plasma concentrations greater than or equal to 0.5 g/dL after infusion with LtEc. Furthermore, statistically significant decreases in blood vessel diameter and increases blood flow from baseline were observed at a plasma concentration of 0.5 g/dL and at all plasma concentrations greater than or equal to 0.5 g/dL after infusion with PEG-LtEc. Statistical differences in blood vessel diameter and blood flow were observed between the two groups at plasma concentrations greater than 0.5 g/dL and at those greater than 1.0 g/dL, respectively.

#### 40–60 μm Arterioles

Changes in 40–60 μm arterioles diameter at various Hb concentrations are shown in Fig 4. Statistically significant decreases in blood vessel diameter from baseline were observed at all plasma concentrations greater than or equal to 0.5 g/dL while statistically significant increases were also observed in blood flow from baseline at plasma concentrations greater than 1.0 g/dL after infusion with LtEc. However, PEGylation resulted in statistically significant changes (increase at 0.5 g/dL and decrease at 1.5 g/dL plasma concentrations) in blood vessel diameter relative to baseline and in blood flow relative to baseline at all plasma concentrations. Furthermore, statistically significant differences in blood vessel diameter were observed between both groups at all plasma concentrations, while statistically significant differences in blood flow between both groups were only observed at plasma concentrations of 0.5 and 1.0 g/dL.

#### 40–60 μm Venules

**Fig 5:**
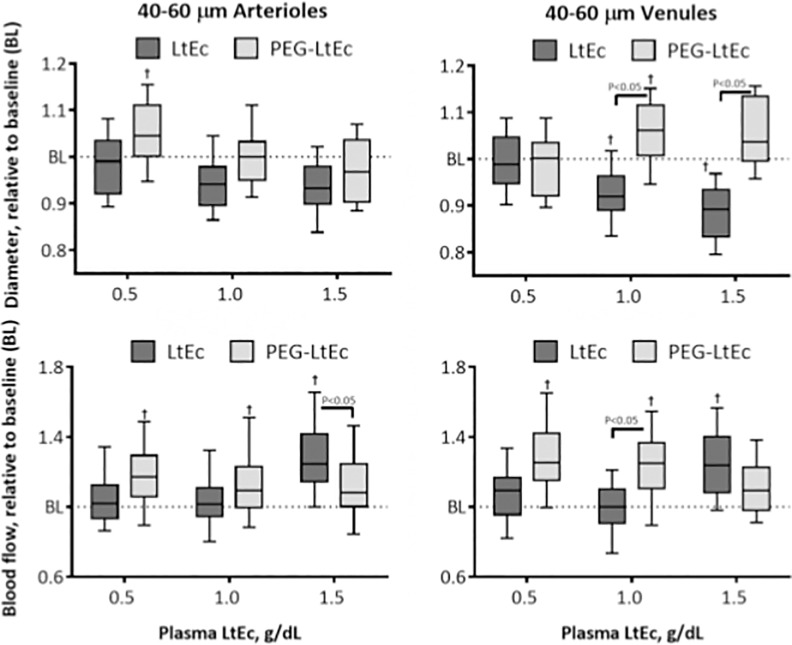
Changes in diameter and blood flow after hypervolemic infusion, of LtEc or PEG-LtEc, for small arterioles and venules with diameters at baseline between 40–60 μm. †, P < 0.05 relative to baseline; ‡, P < 0.05 compared to LtEc. Vessel diameter and blood flow were studied at plasma protein concentration to 0.5, 1.0, and 1.5 g/dL.

Changes in 40–60 μm venules diameter at various Hb concentrations are shown in Fig 5. After infusion of LtEc, statistically significant decreases in blood vessel diameter from baseline were observed at plasma concentrations greater than 0.5 g/dL, while statistically significant increases in blood flow from baseline were observed at plasma concentrations of 0.5 and 1.5 g/dL. However, statistically significant increases in blood vessel diameter from baseline were observed at plasma concentrations greater than 0.5 g/dL and in blood flow from baseline were observed at all LtEc plasma concentrations. Furthermore, statistically significant differences in blood vessel diameters were observed between groups at plasma concentrations greater than 0.5 g/dL, while statistically significant differences in blood flow were observed between both groups at all plasma concentrations.

#### 60–80 μm Arterioles

**Fig 6:**
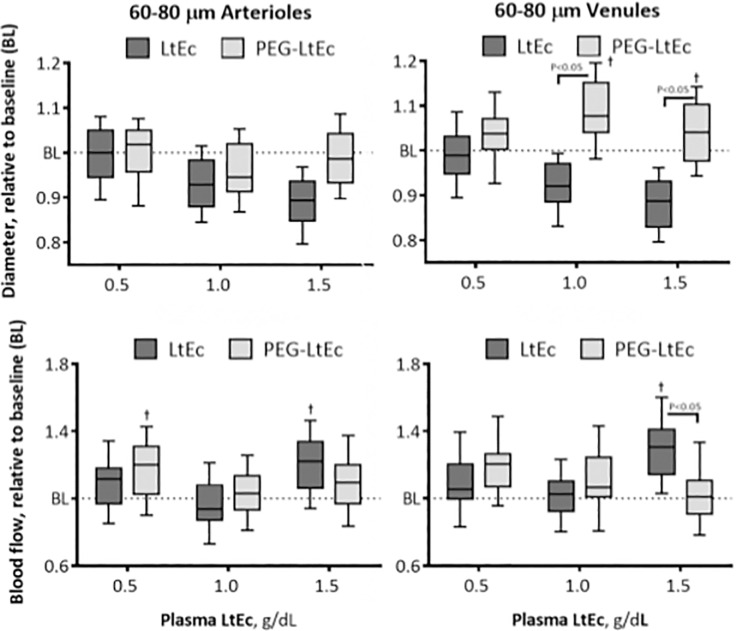
Changes in diameter and blood flow after hypervolemic infusion, of LtEc or PEG-LtEc, for small arterioles and venules with diameters at baseline between 60–80 μm. †, P < 0.05 relative to baseline; ‡, P < 0.05 compared to LtEc. Vessel diameter and blood flow were studied at plasma protein concentration to 0.5, 1.0, and 1.5 g/dL.

Changes in 60–80 μm arterioles diameter at various Hb concentrations are shown in Fig 6. Infusion of LtEc resulted in statistically significant decrease in blood vessel diameter from baseline and increase in blood flow from baseline at plasma concentrations greater than 0.5 g/dL and at plasma concentrations of 0.5 and 1.5 g/dL, respectively. However, infusion only resulted in statistically significant decreases in blood vessel diameter from baseline at a plasma concentration of 1.0 g/dL, and statistically significant increases in blood flow from baseline at plasma concentrations of 0.5 and 1.5 g/dL. Statistically significant differences in blood vessel diameter between groups were observed at a plasma concentration of 1.5 g/dL, while statistically significant differences in blood flow between groups were observed at plasma concentrations of 0.5 and 1.5 g/dL.

#### 60–80 μm Venules

Changes in 60–80 μm venules diameter at various Hb concentrations are shown in Fig 6. After infusion of LtEc, statistically significant decrease in blood vessel diameter from baseline and increase in blood flow from baseline were observed at plasma concentrations of greater than 0.5 g/dL and at plasma concentrations of 0.5 and 1.5 g/dL. However, infusion of the PEGylated protein resulted in statistically significant increase in both blood vessel diameter and blood flow from baseline at all plasma concentrations (0.5, 1.0, and 1.5 g/dL), and at plasma concentrations less than 1.5 g/dL, respectively. Furthermore, statistically significant differences in blood vessel diameter and blood flow were observed between both groups at all plasma concentrations (0.5, 1.0, and 1.5 g/dL).

### Functional Capillary Density

**Fig 7:**
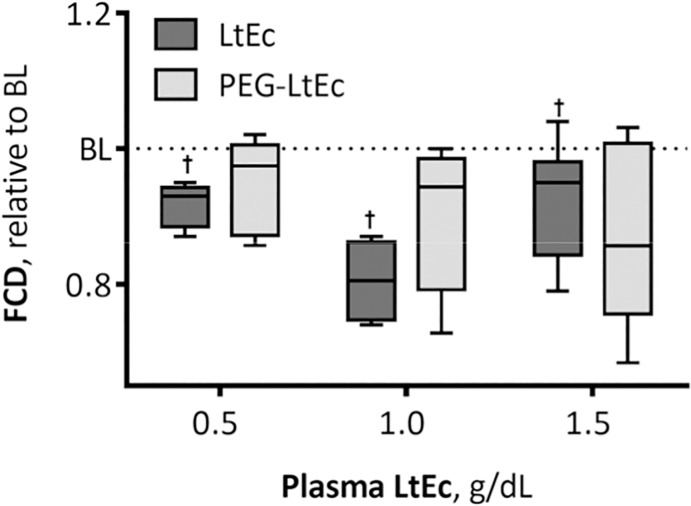
Changes in functional capillary density (FCD) relative to baseline after hypervolemic infusion, of LtEc or PEG-LtEc. FCD was studied at plasma protein concentration to 0.5, 1.0, and 1.5 g/dL. FCD (capillaries per unit of area, cm^−1^) at baseline for LtEc were 119 ± 14, and for PEG-LtEc were 116 ± 16, respectively. †, P < 0.05 relative to baseline; ‡, P < 0.05 compared to LtEc.

FCD after infusion of PEG-LtEc and LtEc is presented in Fig 7. Statistically significant changes in FCD were observed at plasma concentrations less than 1.5 g/dL after infusion with LtEc, while no statistically significant differences in FCD were observed after infusion with PEG-LtEc. Furthermore, no statistically significant differences in FCD were observed between groups at all plasma concentrations.

## Discussion

The principal results obtained in this study confirm LtEc’s potential as an O_2_ carrying therapeutic. PEGylation of LtEc significantly extended LtEc circulation half-life to 66.8 ± 1.6 hours, which was 3.6 times longer than unPEGgylated LtEc. However, PEGylation increased the rate of oxidation of LtEc *in vivo*. As early as 12 hours after infusion, PEG-LtEc is 35% oxidized to metHb, whereas LtEc is only 16% oxidized to metHb. Infusion of PEG-LtEc caused a statistically significant increase in both arteriolar and venular diameters and blood flow relative to baseline. However, no physiologically relevant hemodynamic alterations were observed after infusion of PEG-LtEc systemically or in the microvasculature. In addition, PEG-LtEc preserved MAP, HR, and FCD relative to baseline, and with minor variations from baseline compared to LtEc. No sign of systemic hypertension was observed after infusion of PEG-LtEc. Additionally, no statistically significant differences were observed in volume expansion after infusion between groups infused with PEG-LtEc and those with LtEc. Volumes required to similar increases in plasma concentration were also not statistically difference between both the PEG-LtEc and LtEc groups. Minor increases in COP after PEGylation of LtEc likely did not exert a significant physiological effect due to the already large molecular size of LtEc. Low COP of both LtEc and PEG-LtEc is advantageous over smaller diameter HBOCs. For example, PEGylation of human Hb (PEG-Hb) increases solution COP, leading to volume expansion post infusion, as fluid from the interstitial space moves into the vascular space, reducing the PEG-Hb plasma concentration (i.e. facilitating autotransfusion) and consequently reducing PEG-Hb O_2_ carrying capacity via hemodilution.[26] In addition, all the animals used in this study showed no allergic reactions to PEG-LtEc or LtEc, which confirms that ultra-purification of LtEc eliminates all harmful substances.[14, 15, 18]

Mass spectrum of the purified LtEc confirmed presence of all the LtEc subunits and linkers proteins near their expected MWs; while the mass spectrum of the PEG-LtEc confirms that only a fraction of the LtEc subunits were modified by one or two PEG chains during PEGylation. The non site-specific PEGylation protocol used to generate PEG-LtEc, resulted in a limited homogeneity in the location of the PEG chains. Steric hindrance due to the large size of PEG chains is responsible for limited PEGylation as accessibility by the PEG chains to the subunit was limited. PEGylation using extension arm based on thiol-maleimide chemistry could allow for increasing the number of PEG chains attached to the protein [27]. However, increasing the number of PEG chains on the protein surface still restricts the space available on the protein surface. Consequently, the additional PEG-chains are projected into the solvent phase and are not covering the protein. Mass analysis of LtEc and PEG-ltEc did not detected notable impurities. PEG-LtEc represents a promising technology to increase O_2_ transport when blood is not available for transfusion. Although, the development of PEG-LtEc as a viable alternative to allogenic blood poses many new questions, including efficacy, safety, and clearance. The basis of HBOC toxicity has been poorly understood, since industry research has not been shared among investigators. This study reveals that LtEc is cleared more rapidly from the vascular compartment compared to the PEG-LtEc, which implies that the LtEc degradation begins immediately upon administration. Pilot studies radiolabeling LtEc illustrated no significant accumulation in tissues after 48 hours (data no shown). However, future research exploring the exact mechanism for the removal of the LtEc and PEG-LtEc need to be performed.

Small arterioles are the most sensitive to HBOCs vasoactivity and thus are the active regulators of blood flow. In the analysis of the effects of LtEc and PEG-LtEc on vasoactivity, it is necessary to consider both the type of vessel and size, as the capacity of microvessels to change diameter is different depending on the baseline diameter. Arterioles of 60 to 80 micrometers tend to be more responsive to vasoconstriction; however, when they constrict, the smaller arterioles tend to increase their diameter, as result of myogenic regulation (pressure-induced vascular control). The myogenic regulation is aimed to maintain constant the circumferential stress (so called, hoop stress) resulting from the vascular transmural blood pressure. Therefore, when the large arteriole constricts, the pressure downstream decreases and the diameter of smaller arterioles downstream initially passively follows the reduction in pressure. However, the subsequent myogenic response residing in the smooth muscle itself, relaxes the smooth muscle causing the diameter to increase to normalize the circumferential stress. Therefore, grouping a broad range of microvascular vessels will reduces the capacity to detect changes in diameter in the microcirculation. This study assessed the differences vasoconstrictive response to LtEc and PEG-LtEc at various vessels diameters. To the best of our knowledge, the lack of vasoactivity of LtEc and PEG-LtEct at the various arteriolar and venular diameters, strongly suggest an overall minimal vasoconstrictive response at intravascular concentrations of up to 1.5 g/dL. The changes in arteriole and venule diameter after infusion of PEG-LtEc were mostly vasodilatory from baseline.

Much of the work directed at uncovering the mechanisms of vasoactivity of HBOCs has been conducted using changes in blood pressure and/or systemic vascular resistance. Studies studies, in which HBOCs are infused or used to restore blood volume after blood is removed via hemorrhage, have significant limitations. Changes in blood pressure and/or systemic vascular resistance are in part the result of changes in blood volume resulting from the administration protocol used to infuse the HBOC. Our protocol to administer the LtEc and the PEG-ltEc (exchange isovolemic transfusion) minimally disrupts blood volume, and allows for the quantification of the volume expansion. Therefore, changes in blood pressure can be better interpreted as part of the vasoactive responses or the result of volume expansion. PEG-LtEc did not cause vasoconstriction or hypertension compared to small polymerized Hbs solutions.[18] Instead, PEG-LtEc appeared to slightly relax small blood vessels. The high viscosity of PEG-LtEc (1.9 cP) relative to plasma viscosity (1.2 cP), may also help to produce vasodilation, as plasma viscosity increases endothelial wall shear stress on the blood vessel wall, which stimulates endothelial cell production of vasodilators, including NO and prostacyclin.[28] Previous studies have shown that high viscosity HBOCs improved microvascular function and maintained perivascular NO levels relative to low viscosity HBOCs.[28]

In contrast to most HBOCs, LtEc, even after PEGylation, is not vasoconstrictive. The most accepted mechanism to account for HBOC-induced vasoconstriction is the scavenging of NO through the NO dioxygenation that converts HbO_2_ to metHb and nitrate.[29] The close proximity of circulating HBOCs to the endothelial lining (in contrast to the cell free zone that limits access of RBCs to the endothelial wall) is considered a factor that limits the efficacy of HBOCs with respect to NO scavenging. There are, however, HBOCs that do not induce vasoconstriction.[30, 31] These HBOCs consist of high O_2_ affinity Hbs with large molecular diameters. High O_2_ affinity HBOCs are usually the result of enhanced stabilization of the relaxed (R) quaternary structure of Hb via chemical modification, which is significant as the rate at which deoxyHb converts nitrite to NO is a function of the quaternary structure of Hb [32–37]. The R-state quaternary conformation has much higher nitrite reductase activity relative to the T-state quaternary conformation.[38, 39] The dependence of the nitrite reductase reaction on the quaternary structure of Hb is at least in part due to the difference in the redox potential of the two quaternary state conformations.[40, 41] The R-state conformation has a much lower redox potential, which favors oxidation of a ferrous heme to a ferric heme, as is the case of the nitrite reductase reaction. The intent of the nitrite reductase data presented in Fig 1B is to demonstrate the difference in nitrite reactions between the HbA, LtEc and PEG-LtEc, and to confirm that PEGylation of LtEc preserves the nitrite LtHb reaction, characteristics that can be attributed to the different heme populations of LtEc [42]. The proposed basis for the absence of vasoactivity is a compensatory mechanism in the form of the nitrite reductase reaction that generates NO from nitrite.[10] Overall measured nitrite reductase rate for Peg-LtEc and LtEc indicates a much slower rate as compared to that of HbA, which is consistent with their redox potentials. The rates of autoreduction of the Fe^3+^NO derivatives from the nitrite reductase reaction follow a similar pattern in the PEG-LtEc, and the LtEc is consistent with a functional heterogeneity among the heme sites in LtHb. The functional heterogeneity of the LtEc heme subunits allows LtEc to function both as a stable O_2_ transport protein and as an NO/nitrite-detoxifying agent. This critically important dual functionality could be the result of the driving force for the evolution of subunits with different redox properties. Alternatively, hydrodynamic forces, similar to those that repel particles away from the vessel wall and create the cell-depleted zone, may limit the accessibility of the LtEc protein complex to the endothelium due to its large molecular size.

PEG-LtEc’s longer half-life can be attributed to the relatively hydrophobic polyether backbone, which facilitates extensive hydrophobic interaction, resulting in the formation of clathrate cages of H_2_O molecules around PEG-LtEc. Additionally, an immune response to PEGylation is not observed primarily because the polyether backbone around LtEc present after PEGylation results in steric hindrance, which in turn inhibits recognition of PEG-LtEc by immune cells. [17, 32, 43] Hence, this mechanism slows the rate at which the immune system clears the circulation of PEG-LtEc. Despite met-PEG-LtEc concentrations remaining increased after exchange transfusion, PEG-LtEc concentrations continued to remain stable after 12 hours of circulation. However, this was still found to be lower than that of other HBOCs, which undergo 40% oxidation to metHb in less than 12 hours circulation time. It is speculated that covalent bonding of PEG on specific regions of LtEc have caused either a conformational or electrostatic change that could alter the redox properties of the protein complex, hence altering PEG-LtEc’s autoxidation rate. Furthermore, it was observed that PEG-LtEc appears to have little or no immunogenic effect after consecutive infusions in plasma. As a result, PEG-LtEc appears to be safe, like LtEc, with the greater advantage of a substantially longer half-life.

The short circulation time of LtEc was similar to that observed in a previous study.[14, 18] In addition, our study confirmed that the level of Fe^3+^ (met-LtEc) reached a maximum concentration of 17% in 10 hours, which was much lower than that of other HBOCs.[14] Overall, systemic and microvascular hemodynamic results were consistent with previous studies, in which animals were hemodiluted with LtEc to demonstrate its ability to transport and deliver O_2_.[14] Additionally, previous studies have demonstrated that PEGylation of large proteins decreased the rate of renal clearance and increased the proteins’ biocompatibility, solubility, stability, as well as significantly increasing the circulation half-life.[43]. The long circulatory half-life of PEG-LtEc can be attributed to the hydrophobic polyether backbone, which entropically favors the formation of clathrate cages of water molecules on the LtEc surface.[17, 32] As a result, water cannot hydrogen bond with other nucleophiles present in the circulation, thus increasing the probability of nucleophilic attack of the heme groups, which in turn accelerates metHb formation, explaining the higher oxidation rate of PEG-LtEc relative to LtEc.[10, 32] In addition, cooperativity was significantly reduced, with the Hill coefficient decreasing by 20% from 2.4 to 1.9 for LtEc and PEG-LtEc, respectively. The changes in O_2_-binding properties of LtEc upon PEGylation are like those observed for PEGylated human Hb, in which cooperativity decreases due enhanced stability of the Hb R quaternary state [35]. Considering the stability of the LtEc, this effect is also likely due to the steric effects of the PEG moieties, which prevent the transition between the T and R states, as reported for Hb.

The findings of this study provide evidence to continue development of LtEc as HBOCs. Annually, 15 million RBC units are transfused in the US, while 80 million RBC units are transfused globally. Safe PEG-LtEc can be optimally used to avoid unnecessary transfusions that are costly and increase the risk of transfusion related diseases and immune responses. LtEc can be produced and made available easily at a low cost and large quantities to avoid blood shortages. PEG-LtEc can be rapidly available for transfusion in scenario with limited resources, since there is no need for grouping or typing pre-transfusion. Furthermore, LtEc has the potential to reduce the transmission of yet to be identified pathogens post transfusion, since it is an ultra-purified protein. Unlike previously developed HBOCs, there were no signs of vasoconstriction, systemic hypertension, renal failure, nor allergic reactions in hamsters treated with PEG-LtEc. Additionally, PEG-LtEc is a suitable HBOC for people with religious objections to blood transfusion. In trauma situations, such as resuscitation from hemorrhagic shock, PET-LtEc transfusion increases blood flow, increasing tissue perfusion in addition to just O_2_ carrying capacity.

In conclusion, this study confirms that: 1) LtEc is a potential O_2_ transporter in mammals, 2) intravascular administration of PEG-LtEc has no acute adverse side-effects, 3) PEG-LtEc increases LtEc circulation time, and 4) PEG-LtEc does no elicit vasoconstriction or hypertension, but increases microvascular blood flow. The latter can enhance the benefit of PEG-LtEc’s O_2_ carrying capacity by increasing O_2_ delivery via increased tissue perfusion. In addition, PEG-LtEc’s longer circulation half-life compared to that of LtEc allows for less frequent HBOC administration and extends the benefit of a single administration of PEG-LtEc, until blood for transfusion is available. Additionally, PEG-LtEc can be purified and synthesized at a reasonable cost and its production is scalable. PEG-LtEc’s unique structure and stability in addition to its large molecular size and longer half-life renders it a potent molecule for the development of the next generation of HBOCs. Future studies will be aim to evaluate the O_2_ carrying and delivery capacity of PEG-LtEc.
